# Bone allograft block using demineralized freeze-dried bone ring technique in Indian patients

**DOI:** 10.6026/97320630018577

**Published:** 2022-06-30

**Authors:** Pallavi D Yawale, Kaustubh S Thakare, Priti A Charde, Ashok Kumar Bhati, Aditi A Agrawal, Pooja M Khatri

**Affiliations:** 1 Department of Periodontology, V.Y.W.S. Dental College and Hospital, Amravati, Maharashtra, India; 2 Department of Periodontology, Sharad Pawar Dental College and Hospital, Sawangi (M), Wardha, Maharashtra, India; 3 Department of Preventive Dental Sciences, College of Dentistry, Jazan University, Saudi Arabia; 4 Department of Periodontology, Bhabha College of Dental Sciences, Bhopal, Madhya Pradesh, India

**Keywords:** bone ring technique, alveolar bone loss, DFDBA, bone regeneration

## Abstract

Our study aimed to evaluate the effectiveness of the bone ring technique for ridge augmentation using Demineralized Freeze - Dried Bone Allograft block in Siebert's class II/class III defects along with simultaneous implant placement. A total of 15 partially edentulous patients (16 surgical sites) with Siebert's class II and/or class III defects in the anterior region of both jaws requiring ridge augmentation along with implant placement were selected. Starting from the first stage, surgery (Ridge augmentation+ implant placement) was done in the first month. Then, with continuous follow-ups and radiographic assessment, after 6 months of 2nd stage surgery was done, the implant was loaded with the final restoration. Significant results were revealed with all the parameters other than keratinized gingival and peri-implant mucosa thickness. With the mean bone resorption of 1.22 mm and 1.17 mm at the mesial and distal site at a 6-month interval, the success rate of the bone ring technique was 93.75%. The allograft bone ring technique showed a favorable outcome for the reconstruction of large vertical defects.

## Background:

An extensive periodontal advancement is successfully replacing missing natural teeth using osseointegrated implants [1]. Implant placement nurtures and strengthens the bone, allowing extended mastication and improved aesthetics [1]. Extraction of periodontally compromised teeth/surgical trauma would lead to in sufficient quantity of bone, leading to vertical and/or horizontal defects at the recipient site [2]. Horizontal and vertical bone defects are induced from numerous pathological conditions like periodontal inflammation, pressure from removable prosthesis and physiological resorption in the edentulous jaws, cyst, and tumors [3]. In certain circumstances, alveolar crest bone augmentation may require hard or soft tissue augmentation or both prior to the placement of dental implants [3, 4]. Techniques commonly used for horizontal ridge augmentation are alveolar ridge splitting and expansion, Guided Bone Regeneration (GBR), and Onlay Block grafts (OBG) of autogenic or allogenic origin [5]. For vertical bone augmentation, there are various techniques suggested in the literature including bone block grants, particulate biomaterials, bone in combination with sandwich osteo plastic or membrane technique, distraction osteogenesis. [6] The main drawback with these techniques is that the implant cannot be placed simultaneously and requires around 6 months for healing after bone augmentation, requiring more time for overall treatment. [7] Hence, a novel technique based on Bernhard Giesenhagen's bone ring transplantation procedure was established in 2003 to reduce overall treatment duration and challenges in controlling bone abnormalities. [7] The "bone ring" technique is a surgical methodology that allows bone augmentation and implant placement in one-stage procedure enabling vertical/horizontal augmentation and formation of new bone, there by simplifying the surgical treatment of three-dimensional bone defects. [8] Previously, augmentation was performed with intraorally harvested autogenous bone rings. However, the necessity of a 2nd surgical site, surgical complications associated with unfavorable anatomic structures, the necessity of a large donor site, and patient non-cooperation have led to the use of allogeneic material for bone augmentation [8]. For healing, 6 months of recovery is needed after bone reconstruction, when the vertical and horizontal ridge is insufficient. [9] Therefore, our study aimed to evaluate the effectiveness of the bone ring technique for ridge augmentation using Demineralized Freeze – Dried Bone Allograft block in Siebert's class II /class III defects along with simultaneous implant placement.

## Materials and methods:

A total of 15 partially edentulous patients (16 surgical sites) ranging from 18 - 40 years were selected with Siebert's class II and /or class III defects, needing dental implants for missing teeth replacement, good general and oral health, without any oral or systemic conditions that could adversely affect treatment outcome, and no history of active period on tall and endodontic infection adjacent to graft site were selected from the outpatient department. The institutional ethical board gave the ethical clearance and STROBE guidelines for a human observational study were followed. Routine pre-operative blood investigations were carried out after the patients were informed about the potential risks and benefits, and consent was obtained for the procedure. Preoperative cone beam computed tomography (CBCT) was used to evaluate the surgical site, amount of augmentation required, and the implant's length and diameter to be used based on the regional anatomy. Initial supra and subgingival scaling, was conducted after a thorough examination and diagnosis to achieve plaque control score of < 1, and oral hygiene instructions were given. The Plaque index and Papillary bleeding index was used to evaluate gingival parameters.[10,11] Clinical measurements were recorded using soft tissue index [12] to measure marginal mucosal conditions surrounding oral implants, width of keratinized gingival [13] at the mid-facial aspect of each implant using UNC15 (equinox)® probe, the thickness of peri-implant mucosa [14] by gentle insertion of a sterile Endo reamer using a rubber stopper, approximately 2mm apical to gingival margin until contact of the underlying bone structure, peri-implant probing depth at the buccal, lingual, mesial, and distal aspects of the single-tooth implant by plastic probe (Hu-friedy)®. In addition, implant stability was measured using a noninvasive device called the Osstell device based on resonance frequency analysis principles (RFA). All the clinical measurements were recorded at baseline, i.e., on surgery, 3 and 6 months postoperatively. Radiographic parameters were crestal bone level changes at baseline, 3 months, and 6 months post operatively using RVG. To measure, a tangential horizontal line to the coronal border of the implant was used as a reference. The baseline value was considered 0 at the reference plane. Distance from this line to the most coronal height of crestal bone on proximal surfaces around implants was marked for evaluation of the mesial and distal vertical crestal height of the bone. Values coronal to the reference plane were considered negative and apical were considered positive.[15]

## Surgical procedure:

Before the surgery, 500mg amoxicillin was administered to the patient and instructed to use 0.2% chlorhexidene gluconate once. Following the incision, a full-thickness flap was raised to expose the vertical defect. To establish the right size of the bone ring, a trephine drill with an outside diameter of 6 - 7 mm was used for assessing bony defects. Between rings and adjacent teeth, at least a 1 mm mesiodistal gap was maintained. The ideal implant position was then determined by pilot drilling. For the bone ring, the bed was prepared using a trephine according to the chosen ring size for circular osteotomy at the defect site. The implant bed was prepared through the bone ring with a sequential drilling technique following implant protocol for the myriad implant. The implant was then inserted subcrestally through the bone ring, obtaining primary stability from the local bone and using its crestal portion to keep the bone ring in place, and then a cover screw was placed. Through the bone ring into the native bone, the implant was placed at least 3mm deep in to the local bone. To account for probable resorption, the implant shoulder was placed 1.5 mm below the cranial surface of the bone ring. Then, the edges of the bone ring were smoothened to prevent perforation of the soft tissue; the defect was then covered with osseograft particles. The augmented ridge was then covered with a barrier membrane (Healiguide). Flap closure & suturing was done once the implants were inserted & the cover screw secured. The augmented ridge was covered with a membrane; interrupted sutures were placed using 4-0 vicryl suture material. Postoperative care was followed by 500mg of amoxicillin three times daily for five days. At 3 months follow-up, RVG was taken. At 6months, a second stage surgery was executed. The flap was raised to access the marginal portion of the implant, and the cover screw was replaced with a gingival former. The gingival former was subsequently replaced with a permanent abutment, and implant was loaded with the final restoration. At this stage, again, the stability of the implant was measured. All patients were followed up for 6 months after implant placement, during which patients were evaluated clinically for any infection, pain, soft tissue dehiscence, cover screw exposure, and bone ring exposure.

## Statistical analysis:

The data was entered and analyzed using the Statistical Package for Social Sciences (SPSS) for Windows 26.0. (SPSS, Inc. Chicago, Illinois) Confidence intervals were set at 95%, and ap-value ≤ of 0.05 was considered statistically significant. Repeated measures ANOVA were used to check significance of difference at baseline, 3 months, and 6 months. Further Bonferroni's post hoc analysis was carried out for comparison intra-group group differences.

## Results:

A total of 16 sites in 15 patients (one patient 2 sites) were selected. Out of 16 sites, 5 sites were Sibert class II defects, and 11 sites were Sibert class III defects. Out of 16 implants, 11 were placed in the maxillary anterior region (10 in central incisor region and 1 in the canine region), and 5 were placed in the mandibular anterior region (4 in mandibular central incisor region and 1 in mandibular lateral incisor region). Diameter of implants used was 3.3 mm, 3.5mm, and 3.8 mm, and the length of implants used was 9.5mm, 10mm, 11mm, and 11.5mm. All patients had slight postoperative edema and pain the next day after surgery which subsided completely after 3-4 months. There were no signs of infection, pain, loss of sensation 1 week after the surgery. Out of 16 implants, 15 remained firm and stable throughout the follow-up visits and, after 6 months, received single-unit fixed partial restoration. On the last day of evaluation, all prostheses were functioning. At baseline, the plaque index (PI) was 0.38±0.11, at three months, 0.51±0.21, and at six months, 0.93±0.06. Thus, the difference between PI at baseline and 3months was statistically non-significant, whereas the difference was statistically significant at 3 months and 6 months. At baseline, the PBI was 0.35±0.21, at three months, 0.72±0.17, and at six months, 0.88±0.10. As a result, the difference between PBI at baseline and 3 months was statistically significant, where as the difference between PBI at 3 months and 6 months was statistically non-significant. Soft tissue condition (mucositis score) at baseline was 0.14±0.03, at 3 months, 0.22±0.05, and at 6 months, 0.25±0.07. The difference between the soft tissue condition score at baseline and 3 months was statistically significant, where as difference at 3 months and 6 months was statistically non-significant. The width of keratinized gingiva at baseline was 5.23± 1.17 mm, at 3 months, 4.92±1.18mm, and at 6 months, 4.7±0.96mm. The difference between widths of keratinized gingival at all three intervals was statistically non-significant. The thickness of peri-implant mucosa at baseline was 2.05±0.34 mm, at 3 months, 2.6 ± 0.98mm, and at 6 months, 2.80±1.05mm. The thickness of peri-implant mucosa at baseline, 3 months, and 6 months showed a statistically non-significant difference. Peri-implant probing depth at baseline was 1.79±0.21mm, and at 6 months, 1.24±0.35 mm. The difference between peri-implant probing depth at baseline and 6 months was statistically significant. Implant Stability Quotient (ISQ) score at baseline was 58 ±1.62, and at 6months, 69 ±1.59. Again, the difference between ISQ scores at baseline and 6 months was statistically significant. Radio graphic crestal bone level at 3 months was 0.70±0.06, and at 6 months, 1.22±0.16 at mesial side of the implant. At distal site, the radio graphic crestal bone level at 3-months was 0.69±0.07, and at 6 months, 1.14±0.13. The difference between radio graphic crestal bone level mesially and distally at baseline and 3 months and 3 months and 6months was statistically significant. After 6 months, 15 out of 16 bone rings and implants were successfully integrated without any significant infection and bone loss. Thus, the 6 months survival rate was 93.75%.

## Discussion:

The "bonering" approach enables for a single sitting procedure of bone augmentation and implant insertion. In comparison of the more conventional two-stage augmentation procedure, its advantage is a significant reduction in treatment time. In addition, this technique allows vertical/horizontal augmentation & formation of new bone, which in turn simplifies the surgical treatment of three-dimensional bone defects. [8] All patients in the study group underwent minimally invasive procedures, with graft/bed proximity achieved by preparing the sites with a trephine bur slightly larger in diameter than the bone ring's dimension, allowing the bone ring to fit accurately in its recipient site with adequate stability and maximum bony contact surfaces. The above findings agreed with Marx (2007) study findings, emphasizing on the graft stability significance during the early phases of bone healing and its reflection on early vascularization and graft incorporation. [16] At the time of insertion, all bone ring complexes showed adequate primary stability as measured clinically. Thus, the basic criteria for the success of immediate implants and successful grafting were being fulfilled. [17] All patients demonstrated optimal soft tissue healing at the grafted site and no signs of infection or wound dehiscence, except in one where the bone ring was fractured, and an occurrence of lingual dehiscence occurred observed at the three-month evaluation. There was mobility in the implant, suggesting a failure of the placed implant. This case was excluded from the statistical analysis. The reason for failure could either be the non acceptance of the bone ring or the sharp edges of the ring and thin lingual mucosal coverage. [17] In this study, an allogenic bone ring was used for vertical bone augmentation. In a recent in vivo study conducted by Spin Neto et al. [18] comparing autologous and allogenic bone blocks for lateral ridge augmentation, which proved to be a useful alternative for lateral ridge augmentation and marginal bone level gain, with no significant differences found between the autologous and allogenic groups. Thus, allografts may substitute autogenous bone. For the Plaque index, a statistically significant difference was found in the mean plaque score at 3 and 6 months proving that the patients maintained good oral hygiene in the 3 month study period and gradually decreased at follow-up time. This is in agreement with Weber HP et al. (2000) and Renvert S et al. (2009) study, which showcased similar results explaining the lack of oral hygiene maintenance. [19,20] There was no statistically significant difference in the width of keratinized mucosa at baseline, 3, 6 months evaluation in this study. The wider zone showed more resistance to mastication forces and frictional contact during the oral hygiene procedure. A similar study by Bouri et al. (2008) [13] showed a wider zone of keratinized mucosa (>2mm) having less plaque accumulation and mucosal inflammation. These results can be confirmed as no recession or severe inflammatory changes were noted during the study period. [13] In the present study, the difference in mean peri-implant mucosa thickness at all three intervals was statistically non significant. All the patients of our study had greater than 1mm of mucosal thickness, classifying under thick bio type. Henrickson et al. discovered the same results, demonstrating a substantial increase in peri-implant buccal volume after crown placement. [21] Peri-implant probing depth at baseline was 1.79 ± 0.21, which reduced to 1.24± 0.35 at 6 months re-evaluation in this study. These results may be considered to be in accordance with Schropp et al. study, where the mean peri-implant probing depth at re-evaluation visits was 4mm. [22] Nevertheless, it is acceptable presuming a probing depth of less than 4.0 mm, allowing the patient to undertake self-plaque control and access to skilled peri-implant cleaning. A statistically significant difference was established concerning the implant stability quotient throughout this study period. The results of this study are in accordance with similar results demonstrated by Elnebairy et al. [23] The RVG evaluation demonstrated mean mesial bone resorption of 1.22±0.16mm and mean distal bone resorption of 1.14 ± 0.13 mm at the end of the 6th-month postoperative period. The present data agrees with a study conducted by Crespi et al. [24] who demonstrated that after a 24-month follow-up, mean mesial and distal bone loss was 1.16±0.32mm and1.17±0.41mm, respectively. These findings also match the success criteria for implant treatment stated in the 1st European Workshop on Periodontology consensus report "The criteria of success include average bone loss of less than1.5 mm during the first year after insertion of the prostheses". [25] The bone ring graft success rate was 93.75%, as one bone ring graft in which soft tissue dehiscence was present underwent severe resorption and thus, failed. Other patients saw minimal crestal bone resorption over the 6-month follow-up period, indicating successful incorporation of the bonering graft into the adjacent alveolar bone and adequate osseointegration of the implants into the grafted site. Our results are in accordance with Giraddi et al. study in which success rate of 93.33 % was observed in 14 patients with 15 defects. [15]

## Conclusion:

The present study exhibited the bone ring technique with an allogenic graft as an applicable procedure for bone augmentation and has made implant placement dominant, evident with three-dimensional ridge reconstruction. Also, it was noticed that the width of keratinized gingiva as well as peri-implant mucosa thickness was maintained throughout the study.
